# Butyrate: Connecting the gut-lung axis to the management of pulmonary disorders

**DOI:** 10.3389/fnut.2022.1011732

**Published:** 2022-10-20

**Authors:** Renan Oliveira Corrêa, Pollyana Ribeiro Castro, René Moser, Caroline Marcantonio Ferreira, Valerie F. J. Quesniaux, Marco Aurélio Ramirez Vinolo, Bernhard Ryffel

**Affiliations:** ^1^Laboratory of Immunoinflammation, Department of Genetics and Evolution, Microbiology and Immunology, Institute of Biology, University of Campinas, Campinas, Brazil; ^2^Laboratory of Intestinal Immunology, Institut Imagine, INSERM U1163, Paris, France; ^3^IBR Inc., Matzingen, Switzerland; ^4^Department of Pharmaceutics Science, Institute of Environmental, Chemistry, and Pharmaceutical Sciences, Federal University of São Paulo, Diadema, Brazil; ^5^CNRS, INEM, UMR 7355, University of Orléans, Orléans, France; ^6^Experimental Medicine Research Cluster, Institute of Biology, University of Campinas, Campinas, Brazil; ^7^Center for Research on Obesity and Comorbidities, University of Campinas, Campinas, Brazil

**Keywords:** lung-gut axis, short-chain fatty acids (SCFA), butyrate, inflammation, pulmonary disorders

## Abstract

Short-chain fatty acids (SCFAs) are metabolites released by bacterial components of the microbiota. These molecules have a wide range of effects in the microbiota itself, but also in host cells in which they are known for contributing to the regulation of cell metabolism, barrier function, and immunological responses. Recent studies indicate that these molecules are important players in the gut-lung axis and highlight the possibility of using strategies that alter their intestinal production to prevent or treat distinct lung inflammatory diseases. Here, we review the effects of the SCFA butyrate and its derivatives *in vitro* and *in vivo* on murine models of respiratory disorders, besides discussing the potential therapeutic use of butyrate and the other SCFAs in lung diseases.

## Introduction

The prevalence of pulmonary disorders such as asthma and allergic disease has increased in industrialized countries, which may be partially explained by environmental exposures and changes in lifestyle. The qualitative and quantitative composition of diets is central to health and has a profound impact on the emergence and/or prevention of diseases. In fact, studies comparing different diets and their effects on the composition of the intestinal microbiota have been carried out in recent years. One of the first studies that associated the gut microbiome with different diets in humans occurred when the fecal microbiota of European children was compared with the microbiota of children from a rural African village of Burkina Faso where the diet is based on the ingestion of high amounts of fiber (1). According to this study, African children showed a distinct composition of the intestinal bacterial community with enhanced abundance of Bacteroidetes and lower abundance of Firmicutes, as well as increased levels of SCFAs propionate and butyrate, which was at least four times higher than those of European children. These characteristics were directly linked with the low prevalence of allergies and autoimmune disease in this African population (1). After this, many studies have shown the influence of a Western-style type (WD) diet versus plant-based diets on the intestinal microbiota and the generation of SCFAs (2).

Western-style type (WD) is primarily characterized by high content of animal proteins, fat and refined carbohydrates, which are strongly associated with elevated risks of diseases by promoting body weight gain and changes in energy metabolism and immune system activation (3, 4). In addition, WD has been implicated with negative outcomes on the airway inflammation in chronic obstructive pulmonary disease (COPD) (5). On the other hand, plant-based diets, which are enriched in dietary fibers (DFs), are known to beneficially modulate energy metabolism, systemic immunity and microbiota composition, thus contributing to disease prevention (6, 7). A meta-analysis published by Reynolds et al. (8) found a 15–30% reduced risk of the incidence of several diseases, including heart disease, type 2 diabetes and colorectal cancer in people eating DFs containing whole grains and fruits compared with those with those with the lowest intake (8). In this study, the authors recommend a daily intake of 25 to 29 grams of fibers to prevent chronic diseases and suggested a 15 g increment of whole grains consumed per day to obtain the beneficial effects (8). Regarding the lung context, a study has analyzed data from 1,921 adults retrieved from the National Health and Nutrition Examination Surveys (NHANES) and found that 68.3% of the adults eating more than 17.5 grams of fiber a day (highest fiber group–ingestion of fruits, vegetables, and whole grains) had normal lung function, compared to 50.1% in the group with lowest intake of fiber (< 10,75 grams of fiber a day) (9). Furthermore, only 14.8% of the adults in the highest-fiber group had airway restriction compared to 29.8% in the lowest-fiber group (9).

A diet based on fruits, vegetables, and whole grains was shown to mitigate the inflammatory responses in COPD (5). Furthermore, low fiber diets are associated with reduced diversity of the intestinal microbiota and an imbalanced ratio of metabolites produced by these microorganisms, a process known as dysbiosis, which is involved in the genesis of several pathologies including respiratory diseases (7, 10).

Dietary fibers (DFs) are carbohydrate polymers provided essentially by plant-derived food that can vary in structure, size, and physico-chemical properties (11). Fibers are classified in two main groups: soluble (i.e., gums, fructans, and pectins) and insoluble fibers (i.e., cellulose, hemicellulose, and lignin). Soluble fibers are highly metabolized by the gut microbiota, having relevant effects on composition and production of bioactive metabolites, which can provide a link between microbes and host cells (12). As an example, the consumption of inulin, a type of soluble fiber, has been associated with an increase in beneficial bacteria (e.g., *Bifidobacterium* spp., *Lactobacillus* spp., *Akkermansia muciniphila*, and *Faecalibacterium prausnitzii*) at the expense of potentially pathogenic bacteria (e.g., *Escherichia coli*) in the intestine of adult humans (12, 13). This modulation has a significant impact on metabolic capacity of the microbiota, influencing the profile of metabolites that are produced and consequently, its interactions and effects on host biological functions (6).

Several studies have demonstrated that alterations in the gut microbiome by different dietary approaches can have a significant impact in the outcome of lung diseases (1, 6, 10), besides showing a higher prevalence of lung pathologies in patients with gastrointestinal diseases (14), thus reinforcing the existence of a crosstalk between the intestinal and pulmonary compartments. This bidirectional communication is defined as the gut-lung axis (15), which includes the responses of immune and epithelial cells of both locations, host and microbiota distinct signaling pathways, and the action of bacterial metabolites including the short-chain fatty acids (SCFAs). Therefore, in this review, we aim to discuss the impact of the intestinal microbiota and the action of SCFAs, especially butyrate, on regulating the immune system responses and how this facet of the gut-lung axis may be altered in respiratory disorders. This review compiles the most recent advances in this field, highlighting the gaps and the complexity associated with the different cellular and molecular targets of the microbiota-derived molecules in the context of lung disorders.

## Butyrate: From dietary fibers to cellular effects

Short-chain fatty acids (SCFAs) are small carboxylic acids produced predominantly in the large intestine following fermentation of soluble DFs by the gut microbiota. This class of molecules includes acetate, propionate, and butyrate, with acetate being the most abundant and corresponding to almost half the total production of SCFAs in the colon. Besides carbohydrates, amino acids such as valine, leucine, and isoleucine can also be converted into branched-chain fatty acids, although they contribute to less than 5% of total SCFA production (16–18).

Among these metabolites, butyrate has been shown to have important effects in different aspects of pulmonary diseases such as allergic asthma, COPD, and lung fibrosis (19, 20). Although butyrate can be obtained directly from the diet through the ingestion of dairy products such as butter, it is mainly obtained through the bacterial fermentation of soluble fibers in the colon (19). In humans, most of the Gram-positive bacteria found in the large intestine are butyrate producers, although these are interspersed with other non-butyrogenic species. Butyrogenic bacteria are strictly anaerobic and oxygen-sensitive saccharolytic bacteria from the Firmicutes phylum, including *Ruminococcaceae*, *Lachnospiraceae*, *Erysipelotrichaeceae*, and *Clostridiaceae* (Clusters IV and XIVa) (19, 21–24). Analysis of metagenomic and metatranscriptomic data from human samples has identified an enrichment of butyrate producers, including 17 taxa, primarily members of the *Lachnospiraceae* and *Ruminococcaceae* family along with some *Bacteroidetes*, in 70% of the subjects and in various niches of the gut ecosystem (22). Most of butyrogenic bacteria species are founded colonizing the colon mucus layer in proximity of the intestinal epithelium, which aids the butyrate interaction and physiological, metabolic, and immunologic effects on the host cells (25). In addition, non-butyrogenic bacteria can also influence butyrate formation by the generation of other metabolites such as lactate, which in turns contributes to the acidic gut milieu that favors butyrogenic species in the colon (25).

**FIGURE 1:**
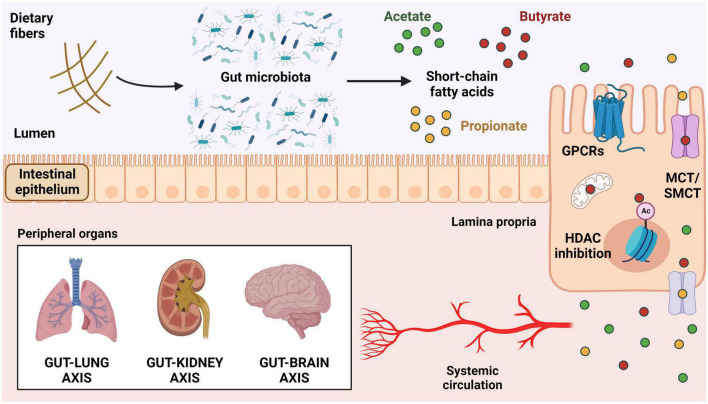
Mechanisms of actions of short-chain fatty acids. Production of SCFAs by bacterial fermentation of soluble dietary fibers in the colonic lumen. These metabolites can activate GPCRs expressed on the surface of intestinal epithelial cells (HCAR2/GPR109a, FFAR2/GPR43, and FFAR3/GPR41) or be internalized by cellular transporters (MCT and SMCT). Once inside the cells, SCFAs can be used in the mitochondria for ATP generation, act in the nucleus as HDAC inhibitors, or be transported outside of the cells into the intestinal lamina propria and subsequently into the bloodstream. Upon reaching systemic circulation, SCFAs can modulate the function of several target tissues, including lungs, kidneys, and brain. SCFAs, short-chain fatty acids; GPCRs, G-protein-coupled receptors; FFAR, free fatty acids receptor; HCAR2, hydroxycarboxylic acid receptor 2; MCT, proton-coupled monocarboxylate transporter; SMCT, sodium-coupled monocarboxylate transporter; HDAC, histone deacetylase.

Once produced, butyrate acts mainly as an energy source to colonocytes and impacts the mucosal homeostasis with effects on the epithelial barrier and the associated immune system (26). Although most of the butyrate is taken up and consumed in the colon, the literature indicates several effects of this metabolite in different peripheral tissues with a significant relevance in the context of lung diseases (27). The systemic effects of butyrate depend on its uptake by intestinal epithelial cells (IECs) and the subsequent distribution of this metabolite through the bloodstream (28). Butyrate is mostly uptaken by IECs by active transport *via* sodium-coupled monocarboxylate transporter 1 (SMCT1, encoded by *SLC5A8*) and proton-coupled monocarboxylate transporter 1 (MCT1, encoded by *SLC16A1*) (Figure 1; 29). Butyrate can also cross the cellular membrane by diffusion when it is protonated in luminal low pH conditions.

Inside the cells, butyrate is largely used to generate ATP in the mitochondria where it is converted to pyruvate and then to Acetyl-CoA to feed the citric acid cycle, but it can also act as a histone deacetylase (HDAC) inhibitor, thus impacting the host epigenome and overall health (26, 30, 31). It has been proposed that other transporters such as proton-coupled monocarboxylate transporter 4 (MCT4, encoded by *SLC16A3*) and proton-coupled monocarboxylate transporter 5 (MCT5, encoded by *SLC16A4*) move the remaining butyrate out of the cells (32, 33), therefore contributing to its passage to the portal circulation into the liver with the other SCFAs (27). In addition, butyrate is also known to bind and activate different G-protein-coupled receptors (GPCRs), such as free-fatty acid receptor 3 (FFAR3, or GPR41), free-fatty acid receptor 2 (FFAR2, or GPR43), and the hydroxycarboxylic acid receptor 2 (HCAR2, or GPR109a) (Figure 1). These receptors are broadly expressed in different tissues and cell types, in humans and animals (2, 34).

**FIGURE 2:**
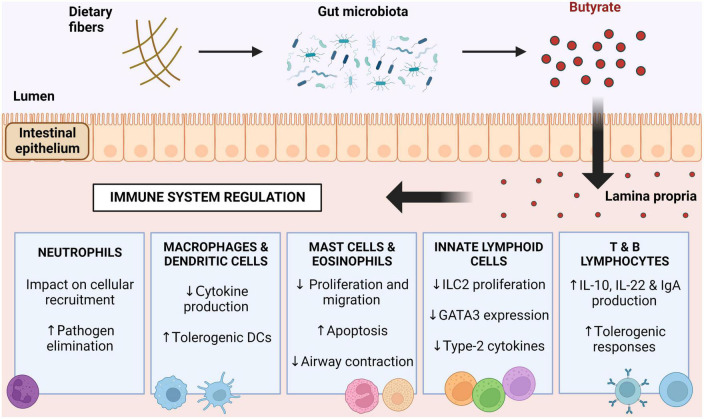
Immunomodulation driven by butyrate. Butyrate produced by butyrogenic bacteria in the intestinal lumen acts on immune cells and regulates their functions. Butyrate induces neutrophil responses to pathogens, reduces cytokine production by mononuclear cells, decreases mast cell, eosinophil and innate lymphoid cell activity, and induces a tolerogenic response in lymphocytes. DCs, dendritic cells; ILC2, innate lymphoid cells type 2; GATA3, GATA binding protein 3; IL-10, interleukin 10; IL-22, interleukin 22; IgA, immunoglobulin A.

Short-chain fatty acids (SCFAs) act on several distinct host organs and tissues including the immune system, brain, bone marrow, and kidneys, exerting a crucial role in the crosstalk between them and the intestinal microbiota, a communication that has relevant implications in the maintenance of the host homeostasis (35). In this regard, alterations in the gut microbiome with changes in the pattern of SCFAs production have local (intestinal) and systemic consequences on physiological and pathological responses. For example, in a dysbiotic state with lower production of these bacterial metabolites, a low-grade systemic inflammation is induced, thus impairing the kidneys functionality and contributing to chronic kidney diseases (36). Additionally, studies have shown that changes in the gut microbiota composition cause a disruption of intestinal-pulmonary crosstalk, which is associated with increased susceptibility to respiratory acute infections and chronic lung diseases (14, 37). Interestingly, the effects of butyrate in the lungs appear to occur indirectly by modulating immune cell function, as this metabolite does not seem to accumulate in the airways, and no significant local production has been described in this tissue (14, 37). Terms such as the “Gut-Lung Axis,” “Gut-Brain Axis,” and “Gut-Kidney Axis” were coined to highlight the relevance of this crosstalk between the gut microbiota and the respiratory, nervous, and renal systems. Butyrate has a significant participation on all these axes through direct or indirect (i.e., through the immune system) actions in different cell types. Hence, in the next section, we will review the main immunomodulatory effects of butyrate described in the recent literature (Figure 2).

## The effects of butyrate/SCFAs on innate immune cells

### Neutrophils

Neutrophils are generally the first cell type to arrive at infectious sites, being a major player that orchestrates the subsequent immune response by the release of several mediators such as cytokines and chemokines. FFAR2 is highly expressed by neutrophils, which makes them very responsive to SCFAs (38). However, divergent results on the effects of the SCFA-FFAR2 activation in neutrophils have been described. *In vitro* activation of FFAR2 by butyrate present in the culture supernatant of the oral commensal *Fusobacterium nucleatum* acts as chemoattractant to neutrophils (39). *In vivo*, oral administration of butyrate has been shown to alleviate intestinal inflammation conditions, but this response can be achieved by modulating neutrophils in different ways depending on the pathological context. For example, in sterile inflammation induced by dextran sulfate sodium (DSS), treatment with butyrate was shown to reduce the recruitment of neutrophils to the colon, lowering the local production of proinflammatory cytokines (40). On the contrary, during inflammation induced by *Clostridioides difficile* infection, the presence of butyrate enhances neutrophil recruitment and their inflammatory activity in the colon, thus improving clinical symptoms (41).

On the contrary, the presence of SCFAs in the site of bacterial infection does not impact neutrophil migration, but it impairs their responses by decreasing their phagocytic capacity and the production of inflammatory molecules. This phenotype does not depend on the GPCRs activation but may involve HDAC inhibition (42). More recently, butyrate was shown to induce the formation of neutrophil extracellular traps (NETs) when added at colonic luminal levels, but not at peripheral blood concentrations (43). Some authors have then suggested that these paradoxical effects of butyrate on neutrophils can be partially explained by the different concentrations that the cells are exposed to. For example, low butyrate concentrations as found in the circulation may activate FFAR2 (and inhibits HDAC), which in turn suppresses neutrophil recruitment and activation, preventing an immune response against commensal microbes and host tissues. However, high concentrations of this metabolite, as found in the colon, may have the opposite effect, favoring neutrophil migration and thereby contributing to elimination of pathogens (44). The discrepancy between the effects of butyrate may also be due to the activation of other cellular mechanisms or to the contribution of the other SCFAs to the biological effects analyzed.

### Monocytes, macrophages, and dendritic cells

Short-chain fatty acids (SCFAs) act as important immunoregulatory molecules preventing the development of exaggerated inflammation (45–47), even though the opposite effect has also been described (48). More recently, one study demonstrated a dual role of SCFAs, in which the innate response is reduced while the adaptive response is promoted, generating effective protection against viral infection in the airways. The authors showed that the high levels of SCFAs induced by high-fiber diets affects hematopoiesis, enhancing the generation of a specific population of patrolling monocytes and alternatively activated macrophages with a limited capacity to produce chemokines. In this sense, fewer neutrophils are recruited to the airways during a flu viral infection, limiting the local inflammatory response and consequently, the immune-associated pathology, while boosting influenza-specific CD8+ T cells to control the viral load (49).

Regarding the actions of SCFAs in macrophages, distinct populations have been shown to be impacted differently by these metabolites. For example, in white adipose tissue the activation of FFAR2 by SCFAs in anti-inflammatory M2-type macrophages increased their production of TNF-α, a response that was not observed in inflammatory M1-type macrophages (50). Moreover, RNA-Seq analysis showed that butyrate induces an antimicrobial signature on macrophages of mucosal sites by the inhibition of HDAC3 (51). Lately, even the systemic inflammatory responses have also been shown to be modulated by butyrate. In a small cohort of obese patients with metabolic syndrome, oral supplementation of butyrate decreased the trained innate immunity of monocytes, which suggests a potential approach for reducing the overall inflammatory status of these circulating cells under certain conditions (52). Corroborating this concept, the literature shows that butyrate is critical to the induction of tolerogenic dendritic cells (DCs), which in turn activate regulatory T cells *via* IL-10 and ALDH1A (53).

A high-fiber diet, *via* SCFAs, induces vitamin A metabolism on CD103+ DCs, which correlates with increased *Foxp3* expression in regulatory T cells (54). Butyrate can also control the expression of costimulatory molecules such as CD40, CD80, and CD83 in DCs, thus limiting their activation by lipopolysaccharides (LPS) (55), besides impacting their chemotactic responses by affecting their responsiveness to CCL19 (56). Furthermore, butyrate profoundly impacts the immune response to allergens by reducing the ability of DCs to migrate to lymph nodes and to prime polarization of the Th2 population (19).

### Mast cells and eosinophils

Mast cells are abundant at mucosa and submucosa sites and are critical in diseases such as allergic asthma, food allergy, colitis, and Crohn’s disease. DFs have a protective effect in animal models of food allergy by controlling mast cells (57). HDAC inhibition by butyrate in murine mast cells suppress their proliferation and production of cytokines (58). In a murine model of colitis, increased levels of SCFAs in the feces were correlated with beneficial effects including improvement of the intestinal barrier and reduction of mast cell degranulation and inflammation (59, 60). More recently, a study showed that OVA-sensitized guinea pig precision cut lung slices incubated with butyrate had a significantly lower release of histamine and decreased airway contraction (61). Butyrate also inhibits degranulation of both human and mouse mast cells, decreasing their production of IL-6 in IgE-and non-IgE, a response that was independent of GPCRs, but that can be linked to butyrate role on HDAC inhibition (61).

Eosinophils are also important players in allergic asthma (62) which may be modulated by SCFAs. SCFAs can affect many eosinophil functions, such as adhesion to the endothelium, migration, and survival. Interestingly, these effects are associated with histone acetylation and are normally independent from GPCR signaling (63), Interestingly, this induction is only observed in eosinophils from allergic donors, while cells from non-allergic volunteers require an extra stimulation by IL-5 to show the same phenotype (63). Inhibition of class IIa HDACs by butyrate also impacts allergic-donor eosinophil migration by decreasing expression of homing chemotactic receptors (19). *In vivo* experiments with intravenous administration of butyrate revealed that this metabolite reduces the number of eosinophils and the concentrations of type-2 cytokines in the bronchoalveolar lavage fluid, thus impacting the allergic response (63). Altogether, these findings further corroborate the immunomodulatory role of butyrate by promoting mucosal tolerogenic responses and protection against allergic disorders (64).

### Innate lymphoid cells

Innate lymphoid cells (ILCs) are regulated by multiple endogenous mammalian cell-derived factors and integrate innate and adaptive immune responses to assist in maintaining physiological homeostasis (65, 66). ILCs are currently divided into five subsets: ILC1, ILC2, and ILC3 (resembling the classic T helper division of Th1, Th2, and Th17), natural killer (NK) cells, and LTi (lymphoid tissue-inducer) cells. This division is based on their distinct transcription factors and production of specific cytokines, although it is well-established that ILCs present high plasticity and can change their phenotype depending on the signals they receive from the microenvironment (67, 68).

The role of SCFAs in modulating the responses of ILCs has been investigated by several studies (69). In this context, due to their location along the gastrointestinal tract, ILC3s have been the most studied subset, with distinct observed results. For example, ILC3s from FFAR2 KO mice have a deficient response to fight against intestinal bacterial pathogens, as well as impaired proliferation (70–72), indicating a positive regulation of SCFAs on ILC3s function. Moreover, butyrate supplementation enhanced IL-22 production by ILCs *via* both HDAC inhibition and activation of FFAR3. The latter effect occurs through activation of the aryl hydrocarbon receptor by hypoxia-inducible factor 1α, a phenotype that support the integrity of the intestinal barrier and to ameliorate colitis (73). However, there are also reports describing inhibitory effects of butyrate on these cells. For example, butyrate can suppress RORγt+ ILC3s and their IL-22 expression in terminal ileal Peyer’s patches *via* HCAR2 activation (74), a receptor not expressed by ILC3s found in the colon (75).

Supporting effects of FFAR2 but suppressing effects by SCFAs have been described also for ILC2s (72). Butyrate administered either by oral or intranasal routes attenuate ILC2-driven inflammatory response in IL-33-and *Alternaria alternata*-induced allergic inflammation, with downregulation of GATA3 expression and ILC2 proliferation, reduction of type 2 cytokine production, and reduced overall airway inflammation and hyperresponsiveness after allergen challenge (76). This suggests that butyrate as a potential therapeutic option for asthma conditions mostly due to its action on inhibiting HDACs (77). Butyrate has also been shown to inhibit pulmonary ILC2 functions by modulating their GATA3 expression and metabolism *in vivo* and *in vitro*, thus protecting against ILC2-driven airway hyperreactivity (78, 79).

## The effects of butyrate/SCFAs on adaptive immune cells

### T and B lymphocytes

Corroborating the anti-inflammatory and tolerogenic roles of butyrate as described above, early studies highlighted the ability of butyrate to promote IL-10 producing T regulatory (Tregs) cells in different organs, thus preventing inflammatory diseases (69). For instance, treatments with butyrate suppressed polarization of pulmonary Th9 cells, attenuating lung inflammation (80). However, chronic elevation of SCFAs levels *in vivo* has been shown to polarize the immune response toward Th1 and Th17, leading to the induction of pathological tissue inflammation (81). Butyrate, *via* HDAC inhibition, increases Foxp3 protein acetylation, resulting in higher Foxp3 levels in Treg cell culture (82). Also, administration of tributyrin, a pro-drug of butyrate, has been shown to reduce several metabolic and inflammatory alterations observed in high-fat diet fed mice (83–85). Tributyrin increased Treg numbers in adipose tissue of obese mice, an effect that was attributed to the activation of HCAR2 and which may be related to the reduction in inflammatory markers at this tissue (84).

In addition to the Treg-inducing effect of butyrate, at high concentrations this metabolite was shown to increase the expression of the transcription factor T-bet on T cells, resulting in IFN-γ production by Tregs and by conventional T cells (86). This dual response might not only depend on butyrate concentrations, but also on the overall conditions in the host. For example, immune tolerance is favored at steady state by SCFAs, with butyrate enhancing the production of IL-10 by Th1 cells *via* FFAR2 activation (87) elevating the production of IL-22 by CD4+ T cells (73), while effector T cell responses are triggered by these metabolites during active immune responses (69).

The effects of SCFAs also extend to CD8+ T cells, with butyrate increasing their expression of IFN-γ, granzyme B, and general cytotoxic function *via* HDAC inhibition (81), as well as optimizing memory CD8+ T cell generation and responses (49, 88, 89). Recent studies demonstrated that SCFAs also have regulatory effects on B cells enhancing plasma cell differentiation and boosting intestinal IgA and systemic IgG responses (84). Butyrate may favor tolerogenic responses by directly enhancing IL-10 producer’s regulatory B lymphocytes (B10) (69, 90), although contradictory phenotypes have been reported, with different doses of butyrate suppressing B10 cells, besides arguing that the previous stimulatory effects were observed due to secondary and indirect effects of this metabolite (91). Nonetheless, these new data reinforce a positive role of butyrate on B lymphocytes under steady state. For example, butyrate causes intrinsic epigenetic alterations on B cells, modulating class-switch DNA recombination and affecting the production of antibodies and autoantibodies, thus preventing harmful responses and helping to maintain the balanced communication between the microbiota and the host (92).

## Effects of butyrate in distinct respiratory disease

Chronic respiratory diseases such as COPD, asthma, and lung fibrosis are among the top ten diseases that affect the respiratory system and are associated with high morbidity, creating a significant health burden. Scientific advances in the treatment of these diseases may reduce this burden and promote health. In the next section, we review the effects of SCFAs and butyrate on respiratory diseases focusing on human or murine studies (Table 1).

**TABLE 1:** *In vitro* and *in vivo* effects of short-chain fatty acids (SCFAs) on distinct pulmonary disorders.

Condition	Fiber type/SCFA	Intervention/dose	Model	Phenotype	References
Chronic obstructive pulmonary disease (COPD)	Cellulose and pectin	Diets with 20% cellulose or pectin 4 weeks of treatment.	Mice model of emphysema	↓ emphysema progression ↓inflammatory responses ↑SCFAs production ↑ microbiota diversity	Jang et al. (141)
	Pectin, fecal microbial transplantation, and mix of SCFAs in the drinking water	Diet with 10% cellulose + 10% pectin. Acetate 76 mM, propionate 29 mM, and butyrate 45 mM. 4 weeks of treatment.	Mice model of emphysema	↓ emphysema severity ↓weight loss ↓apoptosis ↓inflammatory responses ↑ SFCA producers *Bacteroidaceae* and *Lachnospiraceae*	Jang et al. (142)
	Long-term intake of dietary fibers	Data on cereal, fruits, and vegetable consumption	Cohort of 35,339 Swedish women	↓ risk of COPD (30%)	Szmidt et al. (143)
	4-phenyl butyric acid (4-PBA)	0.5 mM of 4-PBA	Human embryonic lung fibroblasts (MRC-5) exposed to 1% cigarette smoke (CS) extract *in vitro*	↓ fibroblasts differentiation into myofibroblasts	Song et al. (98)
Lung fibrosis	Butyrate	1–10 mM of butyrate	MRC-5 human fetal lung fibroblasts treated with TGF-β1 *in vitro*	↓ fibrosis markers ↓mitochondrial elongation in fibroblasts treated with TGF- β1	Li et al. (94)
	Sodium butyrate	Oral administration of 10 mg of sodium butyrate five times a week for 4 weeks.	Lung fibrosis induced by bleomycin in mice	↓ myofibroblast activation ↓Macrophage differentiation in bronchoalveolar lavage fluid	Park et al. (105)
	Butyrate	Intraperitoneal administration of sodium butyrate (100 mg/Kg b.w.) daily for 5 weeks	Pulmonary fibrosis induced by bleomycin in rats	↓ pulmonary inflammation ↓lung fibrosis	Kabel et al. (20)
Allergic asthma	Mix of SCFAs	Acetate 67.5 mM, propionate 25.9 mM, butyrate 40 mM in the drinking water	Ovalbumin model of allergic asthma in mice	↓lung fibrosis ↓IgE and IL-4 production ↓dendritic cell activation and recruitment	Cait et al. (53)
	Inulin	One dose of probiotic yogurt containing 3.5 g of inulin	Analysis of induced sputum from patients with stable asthma	↓ airway inflammation biomarkers ↓immune cell counting, IL-8 and exhaled nitric oxide ↑ expression of FFAR2 and FFAR3	Halnes et al. (144)
	Pectin	30% pectin diet	Allergic airway inflammation induced by dust mite extract (HDM) in mice	↑ circulating SCFAs and ↓ of allergic inflammation	Trompette et al. (44)
	Propionate	Intraperitoneal administration of sodium propionate (1 g/Kg) daily for 2 weeks or 200 mM of sodium propionate in drinking water for 3 weeks	Allergic airway inflammation induced by dust mite extract (HDM) in mice	↓ inflammatory infiltration in airways	Trompette et al. (44)
Lung cancer	Propionate	10 mM of sodium propionate	H1299 and H1703 human non-small cell lung carcinoma	↓proliferation ↑ cell cycle arrest and apoptosis	Kim et al. (129)
	Butyrate	5 mM of sodium butyrate	A549 human lung carcinoma epithelial	↓proliferation and migration ↑ miR-3935 expression	Xiao et al. (128)
Acute respiratory distress syndrome (ARDS)	Butyrate	Intragastric administration of sodium butyrate (25 mg/kg) 1 h before LPS treatment.	Mouse LPS-induced acute lung injury model	↓ IL-1β ↓ TNF ↓ myeloperoxidase ↓ TLR4 ↓NF-κB	Liu et al. (113)
SARS-CoV-2	Mix of SCFAs	Treatment (drinking water) 5 days before SARS-CoV-2 infection and during infection with a combination of acetate (200 mM), propionate (50 mM) and butyrate (20 mM)	Syrian hamsters infected with a sublethal dose of SARS-CoV-2	SCFAs had no effect on clinical and inflammatory parameters	Sencio et al. (135)
	Pectin and mix of SCFAs	Diet with 5% or 30% pectin. Acetate 67.5 mM, propionate 25.9 mM, butyrate 40 mM in the drinking water. Treatments for 2 weeks.	Intranasal infection model in mice and hamsters	↓ viral burdens ↓ SARS-COV-2 entry receptor ACE2 ↑ adaptive responses *via* FFAR2 and FFAR3 in males ↓ coagulation and platelet turnover *via* the Sh2b3-Mpl axis	Brown et al. (139)
	Mix of SCFAs	Mix of SCFAs (acetate, propionate and butyrate with concentrations in the mM range)	Human colon cancer cells (Caco-2) and intestinal biopsies infected with SARS-CoV-2	No effects on anti-viral and inflammatory mediators	Pascoal and Rodrigues et al. (137)

### Chronic obstructive pulmonary disease and cigarette smoke-induced chronic bronchitis with emphysema

Chronic obstructive pulmonary disease (COPD) is a group of progressive inflammatory conditions that lead to irreversible airflow limitation. The fact that we have only limited knowledge about these conditions negatively impacts prevention and treatment options. Tobacco smoking is a major risk factor that affects intestinal microbiota and may affect the production of SCFAs (88–90), but information regarding the effects of SCFAs on COPD is scarce in the literature. Recent studies have shown that COPD patients present impaired intestinal functions (93) besides demonstrating that mice develop elevated lung inflammation and decreased pulmonary functions after receiving microbial transplantation of feces obtained from COPD patients (94) evidencing the relationship between the gut and the lung environments.

Recently, a study in mice investigated the influence of dietary fibers in an experimental model of COPD. According to them, the consumption of a high-fiber diet modulated the diversity of gut microbiota and differentially impacted the generation of SCFAs, bile acids, and sphingolipids, which was associated with attenuated emphysema progression and reduced inflammatory pathology in cigarette smoking-exposed emphysema model (95). Based on the anti-inflammatory properties of butyrate, some studies have aimed to analyze the impact of dietary fibers consumption on the development of COPD in humans. Although with some limitations, these studies revealed that the intake of cereal fibers, and, to some extent, fibers from fruits and vegetables, is inversely associated with the risk of COPD in smokers (96–98). A role of the gut-liver-lung axis has been proposed to impact on smoking-related inflammation: bacterial SCFAs released in the gut, from fiber fermentation, attenuate the innate immune response in the liver, which in turn reduces the lung smoking-related inflammation, ameliorating the symptoms (99). Indeed, cigarette smoke (CS) is known to be the major cause of COPD, with a chronic daily exposure to CS for 6 months resulting in lung inflammation, chronic bronchitis, and emphysema in rodents (100).

Ingestion of a diet enriched in whey peptide was able to attenuate lung inflammation and elastase-induced emphysema in mice, an effect that might be related to increased production of SCFAs in the gut (101). *In vitro*, CS extract was shown to induce human embryonic lung fibroblasts to differentiate into myofibroblasts by causing endoplasmic reticulum (ER) stress, a condition that is associated with fibrosis and that could be suppressed to some extent by the treatment with 4-phenyl butyric acid (4-PBA), a butyrate analog compound (102).

### Lung fibrosis-idiopathic pulmonary fibrosis

**FIGURE 3:**
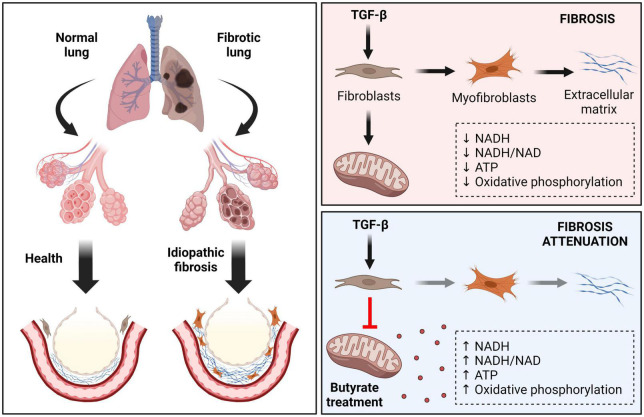
Effects of butyrate in idiopathic pulmonary fibrosis (IPF). IPF is a chronic, progressive, and fibrotic lung disease. Healthy tissue is replaced by an extracellular matrix (ECM) composed by collagen in a TGF-β dependent way. In this condition the alveolar architecture is compromised, leading to decreased lung compliance, disrupted gas exchange, and respiratory failure. TGF-β alters the metabolism and induces pulmonary fibroblasts differentiation, lowering their mitochondrial NADH, NADH/NAD, and ATP levels, as well as oxidative phosphorylation activity. Butyrate acts as a potent antifibrotic factor, restoring mitochondrial activity, and affecting myofibroblast differentiation. TGF-β, transforming growth factor beta; NAD, nicotinamide adenine dinucleotide; NADH, nicotinamide adenine dinucleotide + hydrogen; ATP, adenosine 5’-triphosphate.

Among fibrotic disorders, idiopathic pulmonary fibrosis (IPF) is the most common idiopathic interstitial pneumonia, which is a rapidly progressive and lethal fibrotic disease (103). Recent drugs such Pirfenidone and Nintedanib attenuate disease progression, but currently is no effective therapy for IPF. Limited data are available regarding SCFAs actions, but a recent study has shed some light on this. IPF is characterized by an excessive collagen matrix deposition and extracellular remodeling in a TGF-β dependent manner (104). TGF-β1 alters the metabolism and activates pulmonary fibroblasts, lowering their NADH and ATP levels, the NADH/NAD ratio and oxidative phosphorylation activity (105). Butyrate presented a potent antifibrotic effect by inhibiting mitochondrial elongation in TGF-β-treated pulmonary fibroblasts, increasing their mitochondrial membrane potential and ATP, NADH, and NADH/NAD ratio, affecting myofibroblast differentiation (105; Figure 3). Administration of bleomycin (BLM) has been used as a model of lung inflammation and fibrosis, allowing for investigation of the pathogenic pathways in experimental fibrosis (106–108). Butyrate attenuates BLM-induced lung fibrosis in rats. Animals receiving BLM in combination with butyrate presented a reduction in body weight loss and an improvement on the levels of inflammatory mediators and immune cells in their bronchoalveolar lavage compared to those receiving BLM alone, demonstrating a possible prophylactic role of this SCFA to certain conditions of pulmonary fibrosis (20, 109).

### Allergic asthma

**FIGURE 4:**
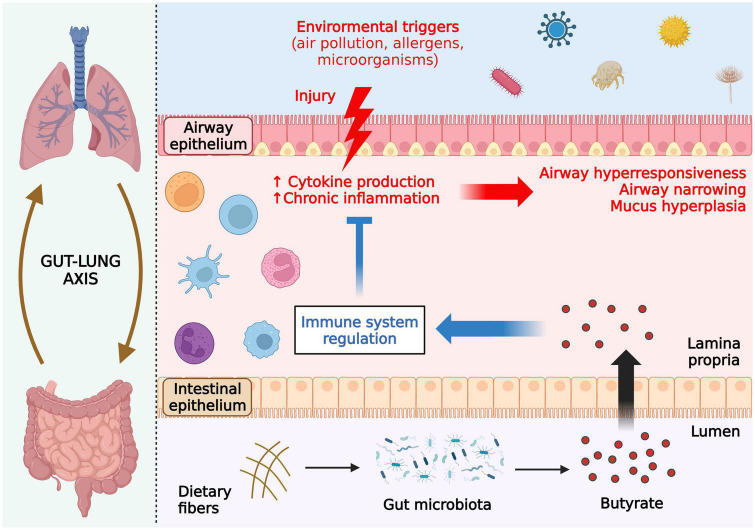
Effects of butyrate in ameliorating allergic asthma. Allergic asthma is associated with sensitization to aeroallergens such as air pollution, bacteria, pollen, and viruses. Exposure to the allergen induces airway epithelial injury, triggering an inflammatory response with enhanced cytokine production and, consequently, airway hyper-responsiveness, narrowing, and mucus hyperplasia. Butyrate produced in the gut reaches the lungs through the bloodstream and regulates inflammatory cells through histone deacetylase (HDAC) inhibition, thus attenuating asthmatic symptoms and pulmonary damage. All figures were created with BioRender.com.

Asthma is an airway chronic inflammatory disorder characterized as a heterogeneous disease. In this review, we focused on the allergic asthma, which is the most common type of this disease (110). Allergic asthma is also associated with sensitization to aeroallergens such as air pollution, bacteria, pollen and virus. The exposure to allergens induces airway epithelial injury and an inflammatory response (Figure 4). It is well-established in the literature that perturbations in the gut microbiota are linked to allergic asthma (14, 111). For instance, the use of antibiotics during pregnancy was found to be associated, in a dose-dependent way, with the severity of asthma in the offspring (108), and several studies have shown beneficial effects of soluble fiber intake and SCFA-producing probiotics for asthma inflammation throughout different stages of life (109, 110). In this sense, SCFAs may influence the development of asthma *via* epigenetic regulation of distinct immune cell populations (19).

Animal studies have also supported the use of SCFAs, including butyrate, to ameliorate allergic asthma (46, 53, 112). More recently, an inverse relationship between asthma and the levels of fecal butyrate, the presence of producing-butyrate bacteria in the gut and the relative abundance of butyrate metabolism enzymes in infants was revealed (113). The lack of genes encoding enzymes for carbohydrate metabolization and butyrate production by the gut microbiota was also shown in infants who develop allergic sensitization later in life (56). Similarly, children with allergic asthma have reduced abundance of *Akkermansia muciniphila* and *Faecalibacterium prausnitzii*, which are known to induce the production of anti-inflammatory mediators through secretion of bacterial metabolites, including butyrate, in the gut microbiota (114). *F. prausnitzii* also presented anti-asthmatic effects in mice by modulating the gut microbiota and altering the levels of SCFAs (115). Other studies have shown that asthmatic children presented lower abundance of *Faecalibacterium* and *Roseburia* spp., reduced levels of fecal butyrate, and increased levels of mite-specific IgE (116). Even a direct intranasal intervention using butyrate showed a protective effect on airway inflammation and fibrosis during allergic asthma *via* HDAC inhibition (Figure 4; 77).

Despite the advances in the field, several important aspects regarding butyrate’s effect in asthma need to be addressed including the identification of its main cellular and molecular targets to prevent the development of asthma (i.e., pulmonary epithelium, resident, or non-resident immune cells) (117) and if butyrate treatment could work together with the other SCFAs (acetate and propionate) or conventional drugs to potentiate their beneficial effects. Since obesity is significantly associated with the development of asthma, worsening asthma symptoms, and leading to poor control of the disease (118) and butyrate attenuates some of the obesity associated alterations, it will be important in the future to investigate if this SCFA can be used for prevention or treatment of obesity-related asthma. Finally, considering that asthma is a heterogeneous disease, it is necessary to further investigate the role of butyrate in each type of inflammation involved with the diseases.

### Lung cancer

Lung cancer is the most diagnosed cancer worldwide and it is implicated in 18.4% of the total cancer deaths (119). Dysbiosis is a common clinical finding in lung cancer patients, suggesting an important role of the gut and lung microbiota in pulmonary carcinogenesis (120, 121). Lung cancer patients present higher levels of *Bacteroidetes, Fusobacteria, Cyanobacteria, Spirochaetes*, and *Lentisphaerae*, and lower levels of *Bacteroidetes, Firmicutes*, and *Verrucomicrobia* in their lung and gut microbiota, respectively (120, 122), thus supporting the link between the imbalanced ratio of Firmicutes and Bacteroidetes with increased risk of cancer development (121, 123). Besides, distinct microbial signature has been described in lung tumor tissues compared to normal samples, with decreased alpha diversity and the presence of specific bacteria such as *Veillonella* and *Streptococcus*, which can be associated with local IL-17 responses and pro-tumorigenic environment (124).

The progression of lung cancer mediated by gut dysbiosis seems to involve mechanisms associated with genotoxicity, systemic inflammation and defective immunosurveillance (125). Furthermore, recent studies indicate that the gut microbiome has a potential to be a novel biomarker for predicting sensitivity and adverse reactions to immunotherapy in lung cancer patients (125). In this context, modulation of commensal microbiota has been shown to impact anti-lung cancer responses in mouse models, with administration of probiotics and fecal microbiota transplantation potentializing the effects of antitumoral drugs (125, 126). Moreover, it was suggested that the altered composition of the gut microbiota could lead to early resistance to immune checkpoint inhibitors, and so, supplementation with bacterial members known to be reduced in lung cancer patients, such as *Akkermansia muciniphila*, could be an option to enhance the action of these inhibitors.

The antitumoral effects of intestinal microbiota metabolites, such as SCFAs, on the suppression of tumor growth, migration and invasion have been widely reported in *in vivo* and *in vitro* studies for several types of cancers. Despite that, studies related to lung cancer are significantly less common (96, 127). Nevertheless, butyrate treatment was shown to inhibit proliferation and migration of A549 human lung carcinoma epithelial cells *in vitro* by upregulation of miR-3935 expression (128). In another study, propionate treatment was able to induce cell cycle arrest and cell apoptosis in the H1299 and H1703 human non-small cell lung carcinoma, besides regulating Survivin and p21 expression, thus suppressing proliferation of these lung cancer cells (129). Altogether, even though the use of SCFAs for therapeutic applications in lung cancer may be beneficial, further studies are necessary to establish and confirm the mechanisms involved in antitumor activity and define optimal doses and routes of administration.

### Acute respiratory distress syndrome

Recent data suggest that butyrate inhibits experimental ARDS (71, 116, 117). Specifically, Liu et al. (113) reported that endotracheal administration of butyrate in a mouse LPS-induced acute lung injury model led to reduced IL-1β, tumor necrosis factor (TNF) alpha and myeloperoxidase in the lung tissue and blood, and diminished TLR4 and NF-κB expression and alveolar wall injury, compared to the LPS treated control group. However, more mechanistic studies are required to increase our understanding of the butyrate effect through investigation of other models of ARDS including viral infection.

In a pertinent example, the ongoing COVID-19 pandemic caused by the SARS-CoV-2 virus, may lead to acute pneumonitis and typical ARDS and death in severe cases (115), caused by cytokine and chemokine overproduction (116). The recruitment and activation of neutrophils and other innate immune cells is a major feature of COVID-19 induced ARDS with the formation of extracellular traps, and damage of the respiratory barrier with edema (130–132). Thus, neutrophils and other myeloid cells are being explored as potential therapeutic targets (133).

The pathways involved in COVID-19 and severe influenza infection in terms of pathophysiology may be shared, but there are also differences between the infections caused by these two viruses (134). Investigations on the intestinal microbial composition and metabolites are of major interest in this case. Altered composition of microbiota has been reported to be correlated with severity of disease in infected hamsters (135) and reduced SCFA and L-isoleucine production in the gut of patients has also been identified (136). However, the treatment with different SCFA concentrations in human biopsies and intestinal epithelial cells infected with SARS-CoV-2 had no effect on production of antiviral and inflammatory mediators (137). These results indicate that the changes in intestinal SCFAs observed on patients with COVID-19 may not be relevant for SARS-CoV-2 effects in the intestine (137). In contrast, a study using a non-human primate model found that the composition and functional activity of the microbiota was altered (138). From these analyses, it is anticipated that altered microbiomes may affect the outcome of acute COVID infection and may be involved in long-COVID sequelae. More recently, one group demonstrated that treatments with a high-fiber diet containing pectin and/or with mix of SCFAs in the drinking water were both able to reduce the levels of SARS-COV-2 entry receptor angiotensin-converting enzyme 2 (ACE2) and, therefore, to reduce viral burdens in intranasal infection model using mice and hamsters (139). According to this study, treatments also increased immune adaptive responses *via* activation of FFAR2 and FFAR3 (but only in males), and reduced coagulation and platelet turnover by regulating the Sh2b3-Mpl axis (139). In this context, in depth analyses of metabolites including SCFAs may emerge a potential new therapeutic in the current and future pandemic.

## Concluding remarks

This review focused on how the gut-lung axis acts on systemic immunity promoting the modulation of aspects related to pulmonary disorders. Butyrate is a key player in the microbiota regulation of immune cells functions. In general, this SCFA contributes to a proper immune response by stimulating key aspects of immunity including antibody production and the recruitment and activation of immune cells, while limiting harmful immune responses. However, butyrate’s effects are complex and context dependent. The beneficial impact of the intestinal microbiota and its metabolites on lung function and on the outcome of diseases such as asthma and COPD is primarily due to the reduction of local and systemic inflammation and, in this context, butyrate has been shown to be a key orchestrator of these responses. Due to the scarcity of effective therapies and considering the potential benefits of microbiota metabolites on respiratory function. Butyrate emerges as a promising agent for the development of therapeutic approaches applicable to pulmonary disorders. However, it should be noted that although studies in the gut-lung axis field are bringing new insights, it is still not possible to determine the best strategies for applications of SCFAs in the clinical management of lung diseases. The main limitations for this application are explained by the fact that most of the studies have been conducted in rodent models, which makes it difficult to extrapolate the data to the human context. In addition, studies performed on humans lack standardization regarding the delivery routes, concentrations of metabolites used, and strategies for modulating the intestinal microbiota. Thus, additional randomized controlled trials are necessary for a better understanding of the mechanisms involved in promoting better respiratory health mediated by the intestinal microbiota and its metabolites. The refinement of these studies may help to enable the use of microbiota metabolites in the clinical context of lung diseases. Finally, recent studies demonstrate that the same strategy used to modulate the lung-intestine axis (i.e., symbiotic, probiotic, or SCFAs) can have a different outcome on allergic airway disease in genetically different mice (110, 140). These findings indicate that the host genetics and its native microbiota are key aspects that need to be considered for the effective use of therapeutic tools that act in the lung-gut axis.

## Limitation of this review

The present review does not address published data on the benefit of local or systemic administration SCFAs or butyrate on IBD, obesity, diabetes, neuro-inflammation, InflammAging, infections, or autophagy mediated processes.

## Author contributions

BR and MV: conceptualization and supervision. RC, PC, and RM: writing – original draft preparation. All authors have editing, read, and agreed on the final version of this manuscript.
